# Transcriptomic Analysis Reveals the Response Mechanisms of Bell Pepper (*Capsicum annuum)* to Phosphorus Deficiency

**DOI:** 10.3390/metabo13101078

**Published:** 2023-10-13

**Authors:** Daizha Salazar-Gutiérrez, Abraham Cruz-Mendívil, Claudia Villicaña, José Basilio Heredia, Luis Alberto Lightbourn-Rojas, Josefina León-Félix

**Affiliations:** 1 Molecular Biology and Functional Genomics, Centro de Investigación en Alimentación y Desarrollo (CIAD), Culiacán 80110, Sinaloa, Mexico; daizha.salazar17@estudiantes.ciad.mx; 2 CONAHCYT-Instituto Politécnico Nacional, CIIDIR Unidad Sinaloa, Guasave 81101, Sinaloa, Mexico; acruzm@conacyt.mx; 3 CONAHCYT-Molecular Biology and Functional Genomics, Centro de Investigación en Alimentación y Desarrollo (CIAD), Culiacán 80110, Sinaloa, Mexico; maria.villicana@ciad.mx; 4 Functional and Nutraceutical Foods, Centro de Investigación en Alimentación y Desarrollo (CIAD), Culiacán 80110, Sinaloa, Mexico; jbheredia@ciad.mx; 5 Genetic Laboratory, Instituto de Investigación Lightbourn, A. C., Jiménez 33981, Chihuahua, Mexico; drlightbourn@institutolightbourn.edu.mx

**Keywords:** bell pepper, differentially expressed genes, phosphorus deficiency, RNA-Seq, transcriptome

## Abstract

Phosphorus (P) is an important nutritional element needed by plants. Roots obtain P as inorganic phosphate (Pi), mostly in H_2_PO^−^_4_ form. It is vital for plants to have a sufficient supply of Pi since it participates in important processes like photosynthesis, energy transfer, and protein activation, among others. The physicochemical properties and the organic material usually make Pi bioavailability in soil low, causing crops and undomesticated plants to experience variations in accessibility or even a persistent phosphate limitation. In this study, transcriptome data from pepper roots under low-Pi stress was analyzed in order to identify Pi starvation-responsive genes and their relationship with metabolic pathways and functions. Transcriptome data were obtained from pepper roots with Pi deficiency by RNASeq and analyzed with bioinformatic tools. A total of 97 differentially expressed genes (DEGs) were identified; Kyoto Encyclopedia of Genes and Genomes (KEGG) enrichment revealed that metabolic pathways, such as porphyrin and chlorophyll metabolism, were down-regulated, and galactose and fatty acid metabolism were up-regulated. The results indicate that bell pepper follows diverse processes related to low Pi tolerance regulation, such as the remobilization of internal Pi, alternative metabolic pathways to generate energy, and regulators of root development.

## 1. Introduction

Plants, being sessile organisms, have a limited range of obtaining nutrients, which is restricted to the place where they are found. The nutritional elements that plants need are diverse, and classified into macro and micronutrients based on the concentrations necessary for their survival. The nutritional elements are called macronutrients if plants require a concentration >0.1% of dry mass [1]. Phosphorus (P) is necessary for plant development and is one of six essential macronutrients (N, P, K, Ca, Mg, and S). P is acquired by plant roots as inorganic phosphate (Pi), mostly in the form of H_2_PO^−^_4_ [2]. An adequate supply of Pi is essential to plants. It is not only a key component of cell molecules, such as nucleic acids, ATP, and phospholipids, but also a crucial regulator in many cellular processes, including energy production, protein activation, and carbon and amino acid metabolism [2]. Also, Pi or its organic derivatives activate significant biochemical processes, such as photosynthesis and respiration [3].

Otherwise, this important nutrient is unavailable because it forms salts with cations, especially aluminum and iron under acid soil conditions and magnesium and calcium under basic conditions. Phosphorus availability is also influenced by ionic strength, concentrations of P and metals, and the existence of competing anions, including organic acids, which usually make the bioavailability in soil scarce. Both crops and wild plants cope with variations in availability or even persistent phosphate restriction [2]. In this context, phosphorus bioavailability is considered one significant limitation of crop production [3]. Interestingly, plants have developed adaptive responses to enhance their well-being during fluctuations in phosphorus reserves [2]. These adjustments include improved topsoil searching by modifying root architecture and growth, through an increased root-to-shoot ratio, increased number and length of root hair; improvement in phosphorus (P) uptake by major activity and number of P transporters; augmentation of P recycling and scavenging for phosphate from intracellular and extracellular organic pools; and enhancement of P economy in metabolic pathways [4,5].

Phosphate deprivation inducible responses are initiated through regulatory pathways involving transcriptional, post-transcriptional, and post-translational regulators by restricting Pi supply. Regulatory pathways arrange the adaptive modifications needed to improve plant condition during variable Pi supply [5]. Recently, 28 phosphate transporters were identified in *Capsicum annuum*, most of them were expressed under P stress, indicating a potential role in Pi mobilization; also, they were expressed in diverse tissues like root, stem, leaf, flower, and fruits, which suggests possible participation in growth and development processes [6]. In recent years, assays related to the transcriptomic analysis of the molecular responses to Pi deficiency have been performed in many species, such as rice, common beans, white lupine, and maize [2,4,7,8]. RNA sequencing has become an effective tool for the analysis of plant genome expression profiles under a diversity of environmental and developmental circumstances [4]. In these crops and model species, the regulatory network working over days of Pi starvation is well characterized, highly conserved, and involves shoot-to-root signaling [9]. This network is likely to be the same in bell pepper. However, mathematical modeling has led to the discovery of a second temporary regulatory process in rice, which is active in the first 3–6 h [10] and can only involve intracellular or local signaling.

To date, however, there are very few reports on phosphorus deficiency in pepper; since it is a limited element, the application of P fertilizer is required to maintain crop yield. According to some estimates, rock phosphate reserves could be depleted in 60–80 years; for this reason, sustainable management of P is necessary. It is important to discover mechanisms that enhance P acquisition and utilize the adaptations to make plants more efficient for acquiring and using Pi [2,11].

In this study, the transcriptome data from pepper roots (*Capsicum annuum* L.) was analyzed in order to identify Pi starvation-responsive genes and their relationship with diverse metabolic pathways and functions. The results will help create a better understanding of the initial responses in bell pepper roots during Pi deficiency.

## 2. Materials and Methods

### 2.1. Plant Material and Growth Conditions

The experiments were conducted using bell pepper (*Capsicum annuum* L.) plants cv. Cannon (Syngenta). The seedlings were germinated in 128-cavity polystyrene trays with a substrate (SOGEMIX PGM GHA Biostimulant, ICAPSA, Culiacán, Sinaloa, Mexico), previously sterilized in an autoclave. The seedlings were individually transplanted into plastic pots with sterile coconut fiber substrate once the third true leaf emerged and kept under greenhouse conditions. The composition of the nutrient solution was as follows: 4 mmol L^−1^ Ca (NO_3_)_2_ · 4H_2_O, 6 mmol L^−1^ KNO_3_, 1 mmol L^−1^ KH_2_PO_4_, 2 mmol L^−1^ MgSO_4_ · 7H_2_O, 100 μmol L^−1^ Na · Fe EDTA, 46 μmol L^−1^ H_3_BO_3_, 9.146 μmol L^−1^ MnCl_2_ · 4H_2_O, 0.76 μmol L-1 ZnSO_4_ 7H_2_O, 0.32 μmol L^−1^ CuSO_4_ · 5H_2_O, 0.0161μmol L^−1^ (NH_4_) 6Mo_7_O_24_ · 4H_2_O, and pH 6.0 [11,12]. After 3 d of adaptation, two groups of five plants were randomly selected and fertilized with phosphorus-deficient nutrition (1 µM P), and the root tissue was collected after 3 h. On the other hand, two groups of five plants with P sufficiency were sampled as the controls. The collected root tissues were immediately frozen in liquid nitrogen and stored at −80° C for later use [13].

### 2.2. RNA Extraction and RNA Sequencing

For this assay, roots of two biological replicates (five bell pepper plants) with phosphorus deficiency and sufficiency were used for total RNA extraction using the TRIzol^®^ methodology (Life Technologies, Carlsbad, CA, USA). Total RNA with absorbance ratios of A_260_/A_280_ ≥ 1.8, A_260/_A_230_ ≥ 1, and RIN values (RNA Integrity Number) ≥ 8 were selected for cDNA library preparation of 150 paired-end reads in the Illumina TruSeq library system. The libraries were sequenced using the Illumina NextSeq-500 platform in the National Laboratory of Genomics for Biodiversity (LANGEBIO) in Irapuato, Mexico.

### 2.3. Bioinformatic Data Analysis

All bioinformatic analyses were performed with free and open-source software. Once the transcriptome sequences were obtained, the raw reads quality was visualized using the FastQC tool (v 0.11.9) [14]. Then, raw reads, including the adaptor sequences and low-quality sequences, were filtered into clean reads using Trimmomatic (v 0.38) [15] with the following parameters: ILLUMINACLIP using the adapter sequence of Truseq3, a minimum mean quality score of 20 (SLIDINGWINDOW:4:20), and a minimum read length of 35 bp (MINLEN:35). Clean reads were aligned to *Capsicum annuum* cv. Zunla-1 genome reference v1.0 (https://www.ncbi.nlm.nih.gov/assembly/GCF_000710875.1/, accessed on 22 June 2020) by hierarchical indexing using HISAT2 (v 2.2.1) [16,17]. The quantification of mapped reads per gene was performed using htseq-count (v 0.9.1) [18], and the differential expression analysis between samples with phosphorus deficiency and sufficiency was performed with the R/DESeq2 package (v 1.32.0) [19] using default parameters, which uses a negative binomial distribution. Those genes with a |Log2-fold change (FC)| > 1 and an adjusted *p*-value < 0.05 were considered as differentially expressed genes (DEGs) [20,21]. The R/EnhancedVolcano package (v 1.10.0) was used to show the up-regulated and down-regulated DEGs. The R/pheatmap package (v 1.0.12) was used to show the expression profiles of DEGs between samples and replicates using normalized counts as inputs.

### 2.4. Real-Time PCR Analysis of Candidate Genes

Total RNA was isolated from pepper roots exposed to low phosphorus with 0 h (control) and 3 h, following the TRIzol^®^ methodology (TRIzol^®^ Reagent from Life Technologies, Carlsbad, CA, USA). A cDNA synthesis was carried out from 1 µg of RNA using the commercial kit Super Script III first strand synthesis kit for RT-PCR (Invitrogen, Carlsbad, CA, USA) according to the manufacturer’s procedure. qPCR was performed using SsoAdvanced Universal SYBR Green supermix (BIO-RAD, Hercules, CA, USA) in mixes and run on a CFX96 Real-Time System (BIO-RAD, Hercules, CA, USA) with the following conditions: a cycle of 30 s at 95 °C, 40 cycles of 10 s at 95 °C and 30 s at 60 °C. A melting curve was performed at the end of the amplification program. To estimate the relative expression, we used the 2^−ΔΔCt^ formula. β-tubulin was used as a reference (housekeeping) gene [22]. Each sample had three replicates.

### 2.5. GO and KEGG Enrichment Analysis

DEGs were mapped to gene ontology (GO) terms in the database (http://www.geneontology.org/). The GO term enrichment was performed in the R/goseq package (v 1.44.0) using the default parameters [23]. To classify DEGs into specific biological pathways, Kyoto Encyclopedia of Genes and Genomes (KEGG) pathway enrichment was performed using KOBAS (v 3.0) (http://kobas.cbi.pku.edu.cn). GO terms and KEGG pathways fulfilling an adjusted *p*-value < 0.05 were considered enriched.

## 3. Results

### 3.1. Analysis of Transcriptome Data

A total of 157,323,048 paired-end raw reads were obtained in this study, with an average of 19,665,381 reads per sample. After filtering with a Phred score > 20 and minimal length > 35 bp, an average of 79% high-quality reads were kept (Table S1). The overall alignment rate of filtered reads against the reference genome ranged between 50.37% and 80.53% (Table S2). Raw counts were obtained for the DESeq2 analysis (Table S3).

### 3.2. Differential Expression Analysis of Pepper Roots

**Figure 1:**
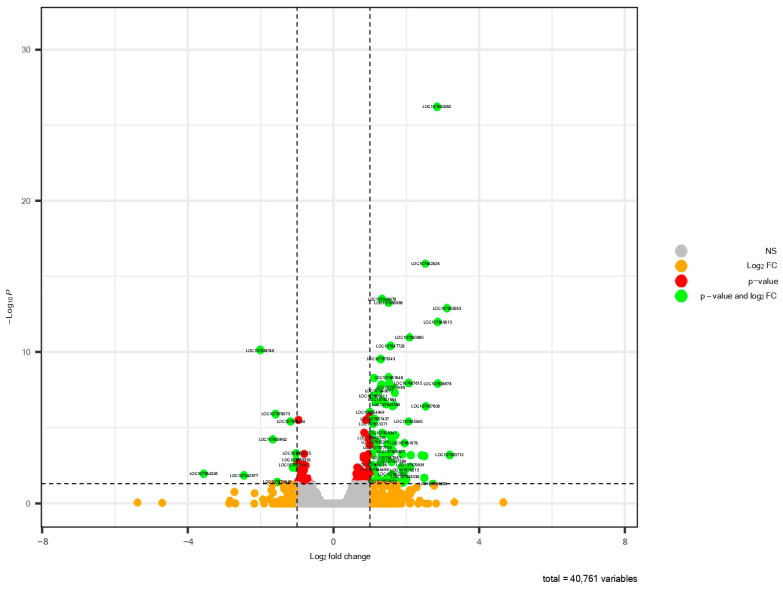
Volcano plot showing differentially expressed genes (DEGs) after 3 h of Pi deficiency in pepper roots. Grey dots are non-significant genes, red dots indicate genes with an adjusted *p*-value < 0.05, orange dots indicate genes with |Log2-FC| > 1, and green dots indicate DEGs.

**Figure 2:**
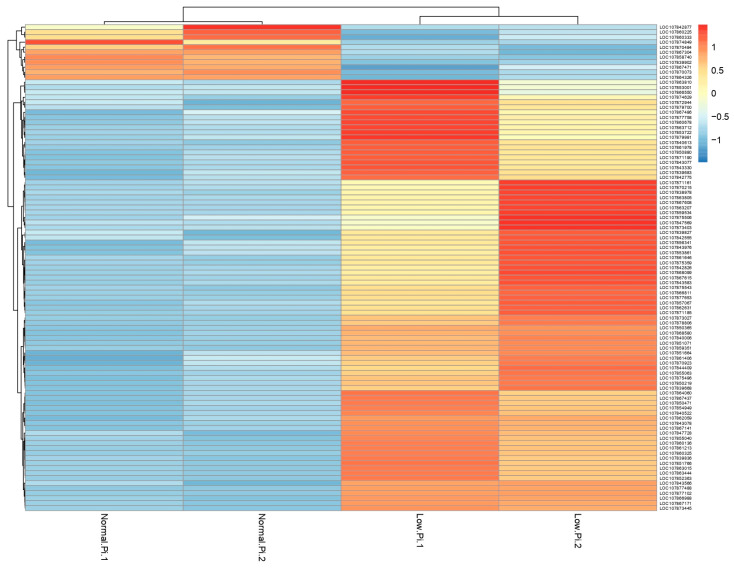
Hierarchical cluster analysis of differentially expressed genes (DEGs) in response to Pi deficiency in pepper roots. The red gradient represents up-regulated DEGs, while the blue gradient represents down-regulated DEGs. Values represent normalized counts transformed to a centered scale.

After 3 h of low phosphorus stress in pepper roots, a total of 97 DEGs were identified (Table 1), which are represented in a volcano plot that shows 86 up-regulated genes and 11 down-regulated genes, with the most statistically significant at the top of the plot (Figure 1). Also, the hierarchical cluster analysis of DEGs showed different expression profiles between the control and the Pi deficiency samples (Figure 2). Six genes (LOC107864060, LOC107867171, LOC107839827, LOC107850880, LOC107873027, LOC107842877) responsive to Pi deficiency were chosen to validate the expression profiles of RNA-Seq data (Figure S1).

### 3.3. GO Term Analysis of DEGs

**Figure 3:**
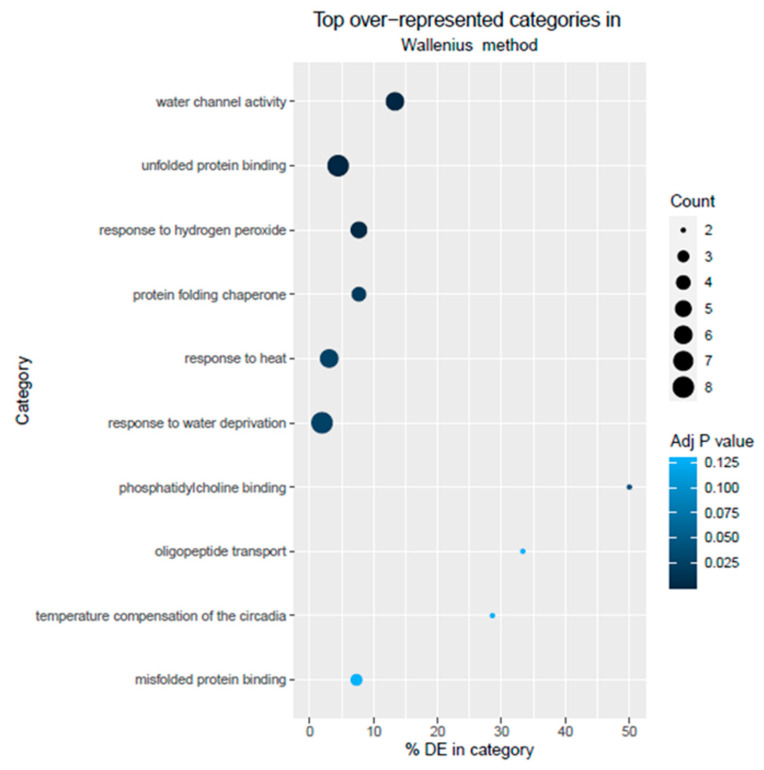
Representative Pi stress-related GO categories. The bubble color indicates adjusted *p*-values (FDR), and the bubble size indicates the number of DEGs in each GO category.

The identified DEGs were mapped with the GO database to be classified into functional categories. Thirty-two GO categories were identified for the down-regulated genes. Of these, six terms were related to cellular component (CC), seven terms were associated with molecular function (MF), and nineteen terms were related to biological process (BP). In the MF category, catalytic activity and union were the prevailing GO terms, while the predominant GO terms for the BP category were cellular process, metabolic process, and reproduction. The cellular anatomical entity, intracellular, and protein-containing complex were the GO terms associated with CC. Also, 22 GO categories were identified for the up-regulated genes, in which cellular process, metabolic process, and response to stimulus were the leading GO terms among the 11 terms in the BP category. Regarding the CC category, three GO terms were identified, including intracellular, protein-containing complex, and cellular anatomical entity. Finally, eight terms were identified in the MF category, including catalytic activity and binding as the prevailing GO terms. To identify plant responses to this abiotic stress, GO enrichments were performed (Table S4), where the DEGs are grouped into over-represented GO categories and represented in Figure 3. Categories with a *p*-value < 0.05 were considered as significantly enriched: water channel activity (GO:0015250), unfolded protein binding (GO:0051082), response to hydrogen peroxide (GO:0042542), protein folding chaperone (GO:0044183), response to heat (GO:0009408), response to water deprivation (GO:0009414), and phosphatidylcholine binding (GO:0031210). These results suggest that low Pi treatment may lead to different responses from the plant in order to counteract the effects of the deficiency.

### 3.4. KEGG Pathway Analysis

To determine the biological functions of the DEGs, the differentially expressed genes were mapped to the KEGG database, and a total of 94 metabolic pathways were identified. Regarding the down-regulated genes, none of the categories were considered enriched, and in the up-regulated, only three KEGG categories were enriched (Tables S5 and S6). The most important pathways were those related to “Porphyrin and chlorophyll metabolism” (cann00860) (Table S6), “Circadian rhythm-plant” (cann04712) (Table S5), “Protein processing in endoplasmic reticulum” (cann04141), “Galactose metabolism” (cann00052), “Endocytosis” (cann04144), “Biosynthesis of unsaturated fatty acids” (cann01040), and “Fatty acid metabolism” (cann01212) (Table S5). These results showed that genes involved in these biological pathways dramatically changed gene expression levels in response to phosphorus deficiency stress. Moreover, from these transcriptomic data, more in-depth analysis was carried out in order to understand the mechanisms of regulation of certain pathways in bell pepper roots under low Pi.

### 3.5. DEGs Associated with Metabolism

Throughout the 3 h of Pi starvation, some metabolic responses were initiated. It was expected to see genes related to plant growth as a metabolic adaptation (down-regulation of protein synthesis and up-regulation of protein degradation). There was only one down-regulated gene, the magnesium–chelatase subunit ChlH. Chloroplastic associated with the metabolism of cofactors and vitamins (porphyrin and chlorophyll) specifically related to chlorophyll biosynthetic process and photosynthesis. There were 13 up-regulated genes; these genes were mainly involved in protein processing, secondary metabolic pathway genes, fatty acid degradation, and carbohydrate metabolism. From these, the 17.8 kDa class I heat shock protein, heat shock cognate 70 kDa protein 2-like, heat shock cognate 70 kDa protein 1, heat shock protein 83-like, E3 ubiquitin-protein ligase MPSR1, small heat shock protein chloroplastic-like, and small heat shock protein (chloroplastic) were involved in protein processing. In addition, related to the biosynthesis of secondary metabolites, the gene 1-aminocyclopropane-1-carboxylate oxidase 1 and L-lactate dehydrogenase B are associated with cysteine and methionine metabolism, methionine being a key metabolite in ethylene biosynthesis. Also, L-lactate dehydrogenase participates in pyruvate metabolism and glycolysis/gluconeogenesis.

Other identified genes were the probable pectinesterase/pectinesterase inhibitor 12, which has catabolic activity and participates in cell wall modification, binding to methylesterases and, therefore, inhibiting their activity. The gene probable trehalose-phosphate phosphatase J (TPP) has trehalose–phosphatase activity and catalyzes dephosphorylation of trehalose 6-phosphate to produce free trehalose and orthophosphate. The gene phosphatidylinositol 4-phosphate 5-kinase 9 takes part in inositol phosphate metabolism, while alcohol dehydrogenase 1 is involved in fatty acid degradation. Another identified gene was the UTP-glucose-1-phosphate uridylyltransferase, which is a key enzyme of the sucrose biosynthesis pathway. Also, thiamine thiazole synthase 1 chloroplastic is involved in thiamine biosynthesis to produce TMP/thiamine/TPP. Finally, ferredoxin, root R-B2-like, is associated with energy metabolism (photosynthesis).

### 3.6. DEGs Related to Transcription Regulation

Fourteen transcription factor (TF) genes were differentially expressed. The expression of thirteen of them was up-regulated and one was down-regulated (Table 1) under Pi starvation. The up-regulated genes included ethylene-responsive transcription factor ERF071-like (root), ethylene-responsive transcription factor ERF054, and ethylene-responsive transcription factor ERF010-like, which were involved in the regulation of gene expression by stress factors and by components of stress signal transduction pathways; they are also related to growth and developmental processes. BTB/POZ and TAZ domain-containing protein 1 were also up-regulated, which might be implicated in Pi metabolism by TF interaction. Also, dnaJ homolog subfamily B member 6-B, dnaJ homolog subfamily B member 6, probable WRKY transcription factor 40, heat stress transcription factor A-7a-like, heat shock factor protein HSF30, transcription factor HBP-1b(c38)-like, multiprotein-bridging factor 1c, and nuclear transcription factor Y subunit B-3-like related to stress response were up-regulated. Finally, the cyclic dof factor 3-like was down-regulated, and this gene is associated with abiotic stress responses and developmental processes.

### 3.7. DEGs Related to Transportation

Genes related to transportation are involved in numerous vital processes in plants, including the transport of macro- and micro-molecules. Ten DEGs-encoding transport proteins were up-regulated in this study. Among these, protein NRT1/PTR FAMILY 6.3-like, phosphatidylinositol transfer protein 1, and protein NRT1/PTR FAMILY 4.3 are related to phosphate transportation. Also, probable aquaporin PIP1-2, probable aquaporin TIP-type RB7-5A, probable aquaporin TIP1-2, probably aquaporin PIP2-4, aquaporin PIP2-1, and aquaporin PIP2-1-like are related to signaling and cellular processes by transporter and channel activity. Finally, the Chloride channel protein, which is an integral component of the membrane and has voltage-gated chloride channel activity, was also up-regulated.

### 3.8. DEGs Related to Stress Response

Several DEGs were related to stress response. The up-regulated genes that respond to abiotic stimulus are the SPX domain-containing protein 1-like, which play an important role in plant adaptation to phosphate starvation since they act as a sensor, and the protein heat-stress-associated 32 is specifically related to heat acclimation. The gene heat shock cognate 70 kDa protein 2-like and heat shock cognate 70 kDa protein 1 are highly conserved molecular chaperones that play essential roles in cellular processes, including abiotic stress responses. On the other hand, heat shock protein 83 and a 17.8 kDa class I heat shock protein that participate as chaperones and folding catalysts, usually in response to exogenous stress, were also identified. The glutathione peroxidase 5 and peroxidase 10 genes are associated with oxidative stress response. The universal stress protein A-like protein participates in stress tolerance (biotic–abiotic), while the E3 ubiquitin-protein ligase MPSR1 is related to proteotoxic stress. The down-regulation of two genes, an ethylene-responsive transcription factor ERF003 and the gene coding for RSI-1 protein, which is related to response to salicylic acid, heat acclimation, plant growth regulation, development, and ripening, was observed.

## 4. Discussion

Differentially expressed genes in pepper roots have different functions in response to low Pi. Nowadays, the absorption capacity of nutrients by plants has become an important topic in different studies, the modifications in the root architecture part [23]. Low Pi availability limits crop growth and yield, particularly in soils (acid-basic) where Pi fertilizers are easily fixed into unavailable forms to crops [24]. Therefore, in recent decades, studies of identification and functional characterization of Pi starvation-responsive genes have emerged to understand the molecular mechanisms that arise in plants exposed to limited Pi availability [11,13,25]. A feature previously reported in *Arabidopsis thaliana* plants is the change in the number and density of lateral roots (LRs) [13]. This is because, as a measure of adaptation to this nutritional deficiency, the plant generates a larger absorption zone in the soil to capture the available phosphorus [26]. Therefore, the regulation of the root architecture establishes an important point of the adaptative response. In this study, the differential expression of 97 genes was identified, including those that encode the transcription factors ERF071 and ERF010, which regulate genes related to the signaling cascade via ethylene pathways, a phytohormone associated with the regulation of root growth, and the formation of root hairs [27]. Related to this, we identified the genes 1-aminocyclopropane-1-carboxylate oxidase and lactate dehydrogenase that participate in the production of methionine, which is a precursor of the ethylene biosynthesis pathway; through its first metabolite, S-adenosylmethionine (SAM), it controls the level of this phytohormone [28]. It has also been observed that ethylene participates in the regulation of the primary root growth under conditions of phosphorus deficiency, which is important for plants since the root provides support to the stem and determines the growth of the plant [29]. This phytohormone interacts with the signaling pathway by auxins, where it has been observed that it induces the growth of lateral roots by mediating the production of auxins and, therefore, the activation of pathways related to this molecule [30].

Also, the up-regulation of the probable transcription factor WRKY 40 and the down-regulation of the magnesium chelatase subunit ChlH were observed. ChlH magnesium chelatase subunit encodes a protein with multiple functions. Still, it is mainly implicated in the regulation of protein synthesis related to photosynthesis and with the perception of ABA. The transcription factor WRKY 40 acts as a negative regulator on ABA-mediated signaling, suggesting a decrease in the energy generation capacity of the plant [31].

Proteins containing the SPX domain are believed to play important roles in Pi signaling networks (detection and transportation) in plants since it is present in a lot of signaling proteins and induced under Pi limiting conditions [32,33]. It is suggested that this domain provides a binding surface for small molecules; in this way, the balance of Pi can be regulated by the interaction with several proteins involved in the uptake, transport, and storage of this nutrient [9,34]. In low Pi conditions SPX genes can influence the transcription of downstream Pi starvation-induced (PSR) genes by regulating PHR activity, possibly via controlling the movement of PHR from the cytoplasm to the nucleus and by decreasing the binding with the P1BS cis-element. It has been described that changes in root morphology, such as the stimulation of root hair growth, are accepted as responses of plant roots to Pi starvation; another important feature of SPX1 is the involvement in regulating root growth and morphology [32].

### Biological Pathways Involved with Stress Response in Pepper Roots under Low Pi

Endocytosis is reported to mediate nutrient uptake, receptor internalization, and regulation of cell signaling. The genes identified in this pathway are PPK (phosphatidylinositol phosphate kinase) related to the development of root hairs and a heat shock protein. The first has been reported to target the endosomal localization of regulatory proteins by binding to PX domains, while the second is a protein that is expressed under stress conditions to protect cells and that has also been reported to interact with aquaporins (AQPs) to regulate the acquisition of nutrients. Since plants do not have a specialized circulatory system, they rely on AQPs to transport solvents, selected solutes, and even reactive oxygen species (ROS); therefore, these proteins have a huge impact during abiotic stress response in plants. Pathways such as glycolysis and gluconeogenesis were also identified, whose metabolites have been reported to participate as signal molecules, mediating multiple physiological responses to stress [35,36]; also, accumulated sugars in bell pepper roots were identified under chill stress as well as organic acids and other metabolites [37]. The availability of sugars activates genes involved in growth and biosynthesis; they primarily affect plant growth by serving as building blocks for anabolic metabolism. They also function as signaling molecules that interact with hormones, including ABA and ethylene, and stress signals are used to control vital processes of growth and development [38].

It has been reported that the accumulation of major soluble carbohydrates (e.g., glucose, sucrose, fructose, trehalose, and maltose) in bell pepper roots is a common response to abiotic stress since these metabolites, in addition to acting as osmoprotectants, help to preserve the osmotic balance they help stabilizing macromolecules under stress conditions. Particularly, the application of trehalose has been studied in maize seedlings under low P conditions, where it improved the growth of shoots and roots as well as lessened oxidative damage [37,39]. This sugar has an important physiological role as an abiotic stress protectant for its ability to scavenge reactive oxygen species, conferring protection to the machinery of protein synthesis [39]. Moreover, sucrose is used to supply the energy required for plant biomass production and also acts as a signaling molecule that coordinates shoots, lateral roots, and root hair development [40,41]. Soluble carbohydrates can also be an energy source for plants unable to perform photosynthesis, which in the case of low-Pi-induced stress, are focusing their machinery on growth processes (remodeling root morphology and architecture) [37,42,43].

Genes related to the metabolism of galactose were identified. According to a recent study, the L-galactose pathway is the prevailing mechanism for ascorbic acid/ascorbate (AsA) synthesis in the leaves and fruits of chili peppers [44,45]. It has also been reported that the rise in the expression of genes is linked to organic acid metabolism, and their exudation under Pi deficiency suggests that organic acids play a significant part in the adaptation to Pi limiting conditions [4,44]. It is known that plants have developed different morphological and physiological modifications to improve Pi acquisition under P-limiting conditions; one of them is the increase in organic acid exudation, establishing a symbiotic relationship with arbuscular mycorrhiza or other beneficial microorganisms. Related to this, roots also secrete flavonoids to delay the microbial degradation of secreted organic acids and to enable rhizosphere Pi mobilization [45]. Interestingly, root exudates of crop plants are known to contain thiamine; this vitamin is an enzymatic cofactor in metabolic reactions that is known to positively influence plant adaptation against abiotic stress via ABA. It was observed that there was an accumulation of thiamine when the plants were under oxidative stress, suggesting an indirect role in enhancing anti-oxidative capacity. Also, thiamine is important for the development and growth of plants since previous studies have shown that this vitamin is essential for the growth and development of roots in many plants [46,47,48].

Alcohol dehydrogenase 1 participates in the previously mentioned routes and the metabolism of fatty acids and is related to energy molecule generation processes (NADH+) through these pathways. It also participates in response to the phytohormone ABA that participates in mediating the development processes, including the increase in the root-to-shoot ratio and root hair density [49]. The hormonal state of the plant affects the root system architecture, and auxins stand out as a key hormone for root development. The circadian clock allows organisms to adapt their growth and development to environmental changes; it is reported that it is rephased during LR development and that its regulation of auxin-related components acts to control the rate of RT emergence. To facilitate organ emergence of LRs, it is necessary to regulate the hydraulic properties by repressing the expression of gene-encoding water channels (aquaporin); however, in other root tissues, the circadian clock regulates aquaporin expression, resulting in diurnal oscillations in root water uptake [50,51].

Related to the circadian rhythm, the GIGANTEA (GI) protein was found, which is related to the regulation of the photoperiodic flowering cycle, and it has been reported that the overexpression of this gene in *Arabidopsis* plants causes early flowering; a mechanism that has been seen as adaptive in another type of stress (drought), which is why it is suggested that under phosphorus deficiency the plant is following this survival mechanism. GI has not only been related to flowering but has also been implicated in regulating various developmental processes such as carbohydrate metabolism, seed dormancy breaking, and wall ingrowth deposition [35,52]. This last process has been proposed as a strategy to increase nutrient exchange, suggesting a potential role of GI in low Pi stress [52].

HEAT SHOCK, BTB/POZ and TAZ domain-containing protein 1 genes were identified. The first one is related to responses to stress, and the other two elements have been reported to participate in the regulation of transcription and degradation in proteasomes, and ubiquitin-protein mediates degradation in proteosomes [35], which suggests that in some way, protein expression is being modulated and folded in order to recycle them to control some essential cellular functions [5,27,31]. In addition, the BTB/POZ domain is a conserved protein–protein interaction motif. Recently, it has been discovered to be crucial in plant development, and it has a role in ABA response, acting as a main component of a signaling network that detects and responds to nutrients, stresses, and hormones [38,53].

Ubiquitination is a very regulated post-translational modification and can result in different outcomes, including stability, localization, activation, and degradation of target proteins. Ubiquitin ligases in plants are well-known to fulfill roles as primary regulators of phytohormone signaling pathways including auxin, ethylene, salicylic acid, brassinosteroid, cytokinin, gibberellic acid, etc. RING-type E3 ligases play important roles in ethylene biosynthesis and distinct aspects of ABA pathways (biosynthesis, transcriptional regulation, and signaling). Also, there is evidence that demonstrates the pivotal function of ubiquitination in regulating plant phosphate acquisition and utilization efficiency [54,55].

## 5. Conclusions

In this study, a global transcriptome analysis was performed to identify genes related to the molecular mechanisms underlying pepper roots responses to cope with low Pi availability. The identification of various transcripts related to transport, Pi homeostasis, growth regulators, and stress-responsive genes indicates that pepper roots follow different processes related to the regulation of the response mechanisms to low phosphorus availability, such as the remobilization of internal Pi, alternative metabolic pathways to generate energy, regulators of root development, and the production of elements that improve phosphorus absorption. This study contributes to understanding the different response mechanisms that pepper roots have to this specific stress. However, further studies are needed to confirm the functions of the identified genes in the distinct processes in order to provide potential target regions for future efforts to develop efficient P pepper plants.

## Figures and Tables

**Table 1:** Differentially expressed genes up-regulated and down-regulated in response to Pi deficiency in pepper roots.

Gene_ID	Gene_Symbol	Log2_FC	P-adj
LOC107864326	glycine-rich RNA-binding protein 2, mitochondrial-like	−3.57005391	0.0107
LOC107842877	ribulose bisphosphate carboxylase/oxygenase activase 1, chloroplastic	−2.46310915	0.0138
LOC107858740	cyclic dof factor 3-like	−2.01495454	0.0000
LOC107839902	protein EXORDIUM-like 5	−1.66993147	0.0001
LOC107870073	magnesium-chelatase subunit ChlH, chloroplastic	−1.59432771	0.0000
LOC107874849	ethylene-responsive transcription factor ERF003	−1.54356028	0.0374
LOC107870484	protein RSI-1	−1.18048035	0.0000
LOC107867304	uncharacterized LOC107867304	−1.11502756	0.0042
LOC107860225	lysine-rich arabinogalactan protein 18	−1.02547235	0.0005
LOC107860333	l-ascorbate oxidase homolog	−1.02069936	0.0014
LOC107867471	protein PHOSPHATE-INDUCED 1	−1.01901492	0.0045
LOC107863712	uncharacterized LOC107863712	1.00184462	0.0000
LOC107855063	protein GIGANTEA-like	1.0094648	0.0006
LOC107838978	heat shock factor protein HSF30	1.02050388	0.0424
LOC107840613	phosphoprotein ECPP44	1.02935247	0.0323
LOC107850365	protein GIGANTEA-like	1.0360357	0.0000
LOC107866550	anthocyanidin 3-O-glucosyltransferase 2	1.04703371	0.0000
LOC107867608	small heat shock protein, chloroplastic	1.04765427	0.0066
LOC107842826	ultraviolet-B receptor UVR8-like	1.05281042	0.0005
LOC107853001	uncharacterized acetyltransferase At3g50280-like	1.05288687	0.0416
LOC107873403	heat shock protein 83	1.06575196	0.0034
LOC107839668	dnaJ homolog subfamily B member 6-B	1.06607451	0.0499
LOC107863207	heat shock protein 83-like	1.06753615	0.0000
LOC107879981	uncharacterized LOC107879981	1.06935719	0.0006
LOC107850880	1-aminocyclopropane-1-carboxylate oxidase 1	1.07409809	0.0028
LOC107867615	small heat shock protein, chloroplastic-like	1.08351024	0.0000
LOC107855040	ethylene-responsive transcription factor ERF054	1.0896119	0.0000
LOC107877102	putative elongation of fatty acids protein	1.10035372	0.0350
LOC107870215	heat shock cognate 70 kDa protein 2-like	1.10550088	0.0001
LOC107843330	phosphatidylinositol 4-phosphate 5-kinase 9	1.11209538	0.0000
LOC107861978	probable WRKY transcription factor 40	1.12111699	0.0055
LOC107875506	17.8 kDa class I heat shock protein	1.12586035	0.0075
LOC107859351	dnaJ protein homolog 1	1.13098799	0.0000
LOC107853722	UTP—glucose-1-phosphate uridylyltransferase	1.14931399	0.0001
LOC107853861	multiprotein-bridging factor 1c	1.15469284	0.0000
LOC107870923	probable pectinesterase/pectinesterase inhibitor 12	1.19701313	0.0202
LOC107839683	universal stress protein A-like protein	1.1972449	0.0001
LOC107862631	BTB/POZ and TAZ domain-containing protein 1	1.20135206	0.0002
LOC107861406	uncharacterized LOC107861406	1.24467066	0.0000
LOC107863805	heat stress transcription factor A-7a-like	1.28616959	0.0020
LOC107861213	uncharacterized LOC107861213	1.29308663	0.0000
LOC107872944	probable aquaporin TIP-type RB7-5A	1.31615269	0.0000
LOC107868099	E3 ubiquitin-protein ligase MPSR1	1.32749478	0.0006
LOC107864060	ethylene-responsive transcription factor ERF071-like	1.33103614	0.0000
LOC107842555	probable glutathione peroxidase 5	1.33549714	0.0322
LOC107847569	dnaJ protein homolog	1.33669919	0.0000
LOC107877488	zinc finger protein CONSTANS-LIKE 5	1.34047692	0.0000
LOC107839827	protein NRT1/PTR FAMILY 4.3	1.35779871	0.0000
LOC107847728	serine carboxypeptidase-like	1.36618804	0.0006
LOC107871190	probable aquaporin PIP1-2	1.43489879	0.0000
LOC107875496	serine/arginine-rich splicing factor SR45a-like	1.45771513	0.0011
LOC107859534	protein HEAT-STRESS-ASSOCIATED 32	1.45968575	0.0216
LOC107861646	dnaJ homolog subfamily B member 6	1.48123286	0.0001
LOC107866988	uncharacterized LOC107866988	1.48592502	0.0001
LOC107878806	nuclear transcription factor Y subunit B-3-like	1.50678499	0.0000
LOC107840522	uncharacterized protein LOC107840522	1.51255887	0.0000
LOC107863810	SPX domain-containing protein 1	1.53228982	0.0249
LOC107857067	protein DELAY OF GERMINATION 1	1.53235936	0.0000
LOC107843566	probably aquaporin PIP2-4	1.5539283	0.0000
LOC107843583	*Capsicum annuum* contig77344	1.55946121	0.0000
LOC107867171	ethylene-responsive transcription factor ERF010-like	1.56569983	0.0004
LOC107851664	ferredoxin, root R-B2-like	1.56750124	0.0000
LOC107856341	thiamine thiazole synthase 1, chloroplastic	1.59743992	0.0018
LOC107877653	temperature-induced lipocalin-1	1.60746717	0.0112
LOC107843078	aquaporin PIP2-1	1.61611701	0.0003
LOC107871161	alkaline/neutral invertase A, mitochondrial-like	1.61885367	0.0000
LOC107873027	alcohol dehydrogenase 1	1.62957467	0.0001
LOC107875543	heat shock cognate 70 kDa protein 1	1.63024785	0.0000
LOC107871185	uncharacterized LOC107871185	1.65724777	0.0000
LOC107843077	aquaporin PIP2-1-like	1.67589881	0.0245
LOC107877758	hemoglobin-2	1.68358102	0.0000
LOC107860678	titin	1.70150059	0.0000
LOC107873445	phosphatidylinositol transfer protein 1	1.78702	0.0387
LOC107875359	peptidyl-prolyl cis-trans isomerase FKBP62	1.86888069	0.0038
LOC107850219	two-component response regulator-like PRR37	1.8876322	0.0006
LOC107867437	protein NRT1/PTR FAMILY 6.3	1.90637911	0.0416
LOC107867141	flavonoid 3′-monooxygenase CYP75B137-like	1.90863477	0.0077
LOC107844409	putative MO25-like protein At5g47540	1.95369314	0.0001
LOC107840006	actin-100	1.96013972	0.0179
LOC107839836	chloride channel protein CLC-c	1.96864421	0.0059
LOC107879700	cysteine-rich repeat secretory protein 38	1.99554005	0.0278
LOC107851071	cell wall/vacuolar inhibitor of fructosidase 1	2.05281355	0.0000
LOC107866811	phosphoprotein ECPP44-like	2.05439524	0.0000
LOC107843976	activator of 90 kDa heat shock protein ATPase homolog	2.0786611	0.0000
LOC107874629	probable aquaporin TIP1-2	2.10954455	0.0037
LOC107863444	alcohol dehydrogenase-like 4	2.12386301	0.0006
LOC107863015	uncharacterized protein At5g23160	2.42136983	0.0007
LOC107860136	uncharacterized LOC107860136	2.49214334	0.0200
LOC107851766	UDP-glucosyltransferase 29	2.4953006	0.0007
LOC107860325	peroxidase 10	2.51720866	0.0000
LOC107852363	probable trehalose-phosphate phosphatase J	2.52472223	0.0000
LOC107842775	protein ENHANCED PSEUDOMONAS SUSCEPTIBILITY 1	2.73066046	0.0491
LOC107868580	serine/threonine-protein kinase SAPK3	2.83899314	0.0000
LOC107867486	l-lactate dehydrogenase B	2.85451109	0.0000
LOC107850471	stearoyl-[acyl-carrier-protein] 9-desaturase, chloroplastic	2.85691431	0.0000
LOC107862059	uncharacterized LOC107862059	3.11160163	0.0000
LOC107854949	early endosome antigen 1	3.18361572	0.0006

## Data Availability

The data presented in this study are available on request from the corresponding author. Data is not publicly available due to privacy.

## Supplementary Materials

The following supporting information can be downloaded at https://www.mdpi.com/article/10.3390/metabo13101078/s1, Table S1: Data processing, quality analysis; Table S2: transcriptome data mapped to the reference genome; Table S3: Raw counts for differential expression analysis; Table S4: GO enrichment analysis; Table S5: KEGG analysis for the up-regulated genes showing the 47 identified metabolic pathways; Table S6: KEGG analysis for the down-regulated genes showing the 47 metabolic pathways related to these genes; Figure S1: Confirmation of the expression pattern of six *C. annuum* genes.
