# Supplementary figures and images for: Transcriptomic Analysis Reveals the Response Mechanisms of Bell Pepper (Capsicum annuum) to Phosphorus Deficiency
Source: Metabolites. 2023 Oct 13;13(10):1078. doi: 10.3390/metabo13101078 (PMC10609356; doi:10.3390/metabo13101078)

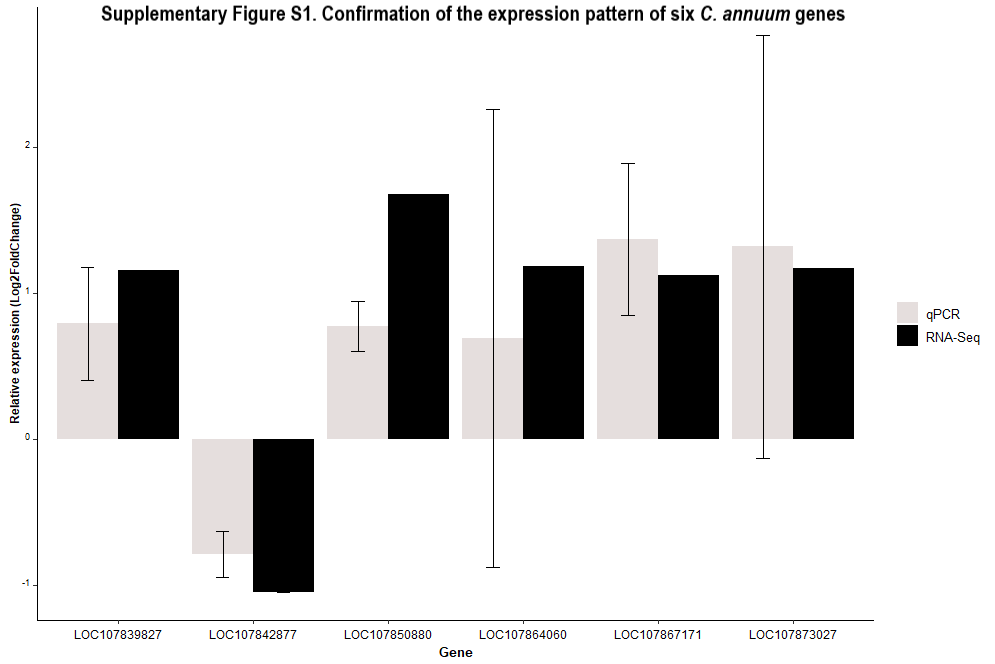

Supplement: Supplementary file 1 [file metabolites-13-01078-s001.zip › metabolites-2531437 - supplementary materials/Figure_S1.tiff]
